# Public Health Screening for Cardiometabolic Risk: Lessons from Advanced Glycation End-Products and ABC Target Achievement in Dalmatian Adults with Type 2 Diabetes

**DOI:** 10.3390/biomedicines13102418

**Published:** 2025-10-02

**Authors:** Josipa Radić, Marijana Vučković, Hana Đogaš, Anders Ødeverp, Marina Grubić, Mislav Radić

**Affiliations:** 1 Department of Internal Medicine, Division of Nephrology, Dialysis and Arterial Hypertension, University Hospital of Split, 21000 Split, Croatia; josiparadic1973@gmail.com (J.R.); mavuckovic@kbsplit.hr (M.V.); 2 Internal Medicine Department, School of Medicine, University of Split, 21000 Split, Croatia; andersodeverp97@gmail.com; 3 Department of Neurology, University Hospital of Split, 21000 Split, Croatia; hana.dogas@gmail.com; 4 Institute for Emergency Medicine of Split-Dalmatia County, 21000 Split, Croatia; marina.grubic123@gmail.com; 5 Department of Internal Medicine, Division of Rheumatology, Allergology and Clinical Immunology, University Hospital of Split, 21000 Split, Croatia

**Keywords:** diabetes mellitus, advanced glycation end-products, cardiovascular risk, arterial blood pressure, cholesterol, HbA1c

## Abstract

**Background/Objectives:** Cardiometabolic risk remains a major challenge in patients with type 2 diabetes mellitus (DMT2). This study aimed to evaluate cardiovascular (CV) risk stratification using advanced glycation end-products (AGEs) measured via skin autofluorescence (SAF) and to assess the achievement of evidence-based ABC targets (HbA1c, blood pressure, low-density lipoprotein (LDL) cholesterol) in adults with DMT2 in Dalmatia. **Methods:** In this single-center cross-sectional study, 251 adults with DMT2 were stratified by CV risk based on SAF measured AGE levels. Clinical, biochemical, and anthropometric data were collected, including ABC goal attainment and medication use. Statistical analyses compared groups and explored predictors of ABC target achievement using regression models adjusted for clinical factors. **Results:** Only 17.5% of participants achieved all three ABC goals, indicating suboptimal cardiometabolic control. Those with elevated CV risk had higher hip circumference and lower diastolic blood pressure. Use of sodium-glucose cotransporter 2 (SGLT2) inhibitors was positively associated with ABC goal achievement in patients with prior CV or cerebrovascular events, while higher body mass index was negatively associated. SAF measured AGE levels correlated with cardiometabolic risk but showed no significant differences across LDL cholesterol or other traditional markers. **Conclusions:** SAF AGE measurement shows potential for CV risk stratification in DMT2 beyond traditional factors. The low rate of ABC goal attainment highlights the need for intensified individualized management incorporating novel biomarkers and therapeutics like SGLT2 inhibitors. Further prospective studies are needed to validate these findings and improve prevention of cardiovascular complications in DMT2.

## 1. Introduction

Diabetes mellitus (DM) is a chronic disease of persistently elevated blood glucose due to abnormal beta cell production of insulin and/or insulin action [1,2]. Diabetes has over the years turned into a modern epidemic and global public health issue, representing one of the most prevalent chronic diseases worldwide [3]. The International Diabetes Federation’s (IDF) last update on the diabetes epidemiology of clinically confirmed cases states that 1:9 adults aged 20–79 years live with DM and 4:10 are unaware of having the condition [4]. The World Health Organization estimated that over 800 million adults are living with DM, diagnosed or undiagnosed, where 90% is attributed to type 2 DM (DMT2) [5]. DMT2 is therefore one of the most common metabolic disorders to date [6].

The strongest etiologic pathogenic link to DMT2 is through an obesity genetic component influenced by the modern world of multifactorial environmental exposures, sedentary lifestyle, and rising obesity rates [7]. DMT2 is also a highly comorbid disease where around 70% of patients have hypertension and over 50% have some sort of dyslipidemia, each carrying its own risk of complications [8]. To mitigate the cumulative risk of complications, the American Diabetes Association (ADA) made an ABC protocol, which is the current gold standard guideline for monitoring adults with DMT2 [9,10,11]. It consists of glycemic control, blood pressure (BP) management, and lipid regulation through the defined ABC goals: A—hemoglobin A1c (HbA1c), < 7%, B—BP < 140/90 mmHg and C—Low-Density Lipoprotein Cholesterol (LDL-C) < 2.6 mmol/L [12,13,14,15,16]. The strong link between DMT2 and arterial hypertension (AH) is more pathophysiologically intertwined than coincidental [17,18,19]. Insulin resistance, hyperinsulinemia, and chronic hyperglycemia induce elevated BP through a complex interplay [17,19]. AH is a major contributor to cardiovascular (CV) morbidity and mortality in DMT2 patients [20]. Since long-term outcomes of AH include vascular remodeling, there is an increased risk of stroke, myocardial infarction, and renal disease [21]. Hypercholesterolemia, more particularly elevated LDL-C, amplifies CV risk in DMT2 patients through a state of chronic inflammation and atherosclerosis [22]. Insulin resistance and chronic hyperglycemia decrease High-Density Lipoprotein Cholesterol (HDL-C) and increase LDL-C levels in the blood [22,23]. This induces a proatherogenic milieu for macrovascular changes with high risk for fatal cerebrovascular (CBV) events [24]. A long-term follow-up study on the cumulative effect of the comorbid nature of the disease and complications show the importance of intensive and extensive monitoring to reduce the continuum of morbidity [25]. However, the complex nature of the comorbid disease complications has shown that only a minority of patients reach the three ABC targets simultaneously, even on optimal therapy [26,27].

Advanced glycation end-products (AGE) hold great potential in monitoring DMT2, as they have been shown to contribute to the pathogenesis of chronic diabetic complications [28]. Importantly, hyperglycemia mimics the natural process of aging through faster aggregation and deposition of AGE in tissues [29]. A measurable proportional increase of AGE is said to start early in the occurrence of hyperglycemia [30]. Increased AGE and activation of intrinsic cellular cascades induce the production of its receptor (RAGE), which escalates the inflammatory process behind diabetes complication pathogenicity [31]. AGE, as opposed to serum concentration, can also be measured non-invasively in the skin with autofluorescence techniques (SAF) as a predictor of microvascular complication development and mortality [32]. The levels of SAF-measured AGE and macrovascular cardiovascular disease (CVD) severity assessment have also been well established [33]. Furthermore, SAF shows promise as an early screening tool for patients with risk factors of developing DMT2 [34].

Various studies have correlated high serum AGE or high skin AGE with poor diabetes control of the HbA1c, BP, and lipid panels [34,35,36]. Recent evidence shows a linear relationship between serum AGE levels and ABC protocol parameter levels in poorly regulated diabetic patients [37]. Rezaei et al. revealed in a cross-sectional study that DMT2 patients with AGE levels above 73.9% in serum have a 2.2 times higher likelihood of not reaching the ABC target [37]. Recent evidence indicates that SAF-measured AGE correlates strongly with both microvascular (such as diabetic retinopathy and nephropathy) and macrovascular endpoints, including CVD and stroke risk [12,13]. For example, independently of hyperglycemia, skin accumulation of AGE contributes to the progression of diabetic nephropathy [38], and elevated skin AGE has been independently associated with carotid intima-media thickness in DMT2 patients [39].

Despite the implementation of the guidelines and novel pharmacotherapy modalities, there is a persistent proportion of DMT2 patients who are sub-optimally regulated [26]. The need to manage multiple parameters induces a higher chance of sub-optimal adherence, resulting in a higher burden on patients and healthcare workers [11]. By integrating SAF-measured AGE into routine monitoring protocols, clinicians may gain an early window into the cumulative metabolic burden of hyperglycemia and dyslipidemia, potentially enabling tailored therapeutic adjustments that preempt irreversible vascular damage.

Therefore, the aim of this study is to assess ABC target achievement and its association with skin AGE levels in participants with DMT2. Given their direct association with pathophysiological changes in tissues, we hypothesize that skin AGE might provide additional or earlier insights into metabolic dysregulation.

## 2. Materials and Methods

### 2.1. Study Design

This cross-sectional study was conducted in the Division of Nephrology, Dialysis, and Arterial Hypertension, Department of Internal Medicine, and the Division of Medical Laboratory Diagnostics at the University Hospital of Split, Croatia. The research took place between November and December 2023, coinciding with World Diabetes Day, as part of a public call for all Dalmatian patients with diabetes.

The study protocol was approved by the Ethics Committee of the University Hospital of Split on 27 November 2023 (Number: 2181-96147/01/06/LJ.7.-23-02, Class: 500-03/23-01/225). Before participation, all individuals were thoroughly informed about the study’s purpose and procedures, and written consent was obtained. This study takes a closer look at AGE and the above-mentioned ABC targets within this population as part of a sub-analysis, expanding on findings from previously published papers [40,41].

### 2.2. Population

A total of 288 participants were initially screened during the public call, including some minors who were accompanied by their parents. The inclusion criteria required participants to have a confirmed diagnosis of DMT2 and be at least 18 years old. Individuals diagnosed with DM type 1 (DMT1) or those under 18 were excluded. Among the exclusions, 36 participants had DMT1 (31 of whom were over 18 and 5 under 18), and an additional 5 participants were excluded due to being minors. Also, one participant was excluded due to a lack of AGE measurement.

**Figure 1:**
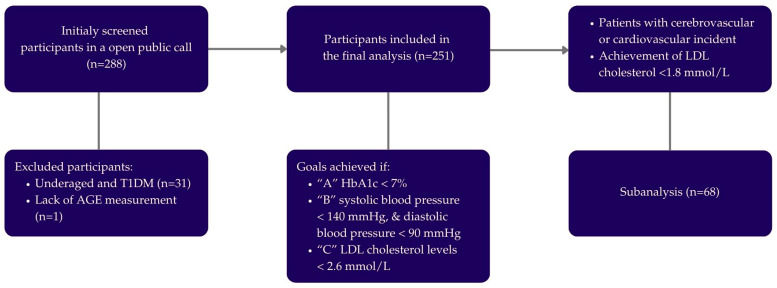
Study protocol.

Ultimately, 251 participants (130 women and 121 men) with DMT2 were included in this study. Their diagnosis had been previously confirmed by a family medicine doctor or endocrinologist, following the diagnostic criteria established by the ADA. This study also included a sub-analysis of AGE, expanding upon results from previously published papers [42,43]. To further comprehend the CV risk in this population, the participants were divided according to achieved glycemia (A), arterial BP (B), and LDL-C (C) goals. If hemoglobin A1c (HbA1c) was lower than 7%, “A” goal was considered achieved. If systolic BP was lower than 140 mmHg and diastolic BP was lower than 90 mmHg, “B” goal was considered achieved. If LDL-C levels were lower than 2.6 mmol/L, “C” goal was considered achieved. Also, we performed a sub-analysis of 68 DMT2 patients with high CV risk who had CV and/or cerebrovascular incidents in their medical history. The study protocol is depicted in Figure 1. For clarity and context, Supplementary Table S1 compares the uniform ABC thresholds used in this study with the latest individualized targets recommended in the ADA and European Society of Cardiology (ESC) guidelines. This table illustrates that patient-specific targets for HbA1c, blood pressure, and LDL-C management are emphasized in current practice.

### 2.3. Medical History, Clinical and Laboratory Parameters

A team of physicians, medical students, and dietitians conducted a lifestyle questionnaire under the supervision of certified medical professionals. This questionnaire collected key participant information, including age, gender, medical history, prescribed medications, and lifestyle habits such as smoking history and duration for current smokers. Participants were also asked about previous consultations with nephrologists or endocrinologists and whether they had ever received nutritional advice. Additionally, we documented any coexisting conditions, including DMT2 treatment duration, AH, kidney disease (KD), CVD, CBV disease, and other chronic illnesses.

On the day of the study, the participants provided a first-morning urine sample and underwent routine blood sampling. Laboratory analyses were performed at the Laboratory of Medical Diagnostics and Biochemistry at the University Hospital of Split, Croatia, using methods described previously [40,41]. The laboratory parameters measured included white blood cell count (WBC; ×10^9^), red blood cell count (RBC; ×10^12^), hemoglobin (Hb; g/L), mean corpuscular volume (MCV; fL), mean cellular hemoglobin (MCH; pg), mean cellular hemoglobin concentration (MCHC; g/L), hematocrit (Htc; L/L), red cell distribution width (RDW; %), neutrophils (%), lymphocytes (%), monocytes (%), basophils (%), and eosinophils (%). Biochemical markers included glucose (mmol/L), hemoglobin A1c (HbA1c; %), triglycerides (Tg; mmol/L), HDL-C (mmol/L), total cholesterol (mmol/L), LDL-C (mmol/L), and creatinine (μmol/L). Kidney function was assessed using the estimated glomerular filtration rate (eGFR) calculated with the Chronic Kidney Disease Epidemiology Collaboration (CKD-EPI) equation. Urine samples were analyzed for creatinine (mmol/L), albuminuria (mg/L), and albumin-to-creatinine ratio (ACR; mg/mmol). KD was defined as eGFR < 60 mL/min/1.73 m^2^ or albuminuria > 3 mg/mmol. Goal LDL-C values for general participants were under 2.6 mmol/L, while for those participants who had high CV risk were under 1.8 mmol/L. This comprehensive dataset allowed for a detailed evaluation of participants’ health status, providing insights into metabolic, CV, and renal function.

### 2.4. Body Composition and Anthropometry Measurements

We assessed body composition using the MC-780 Multi-Frequency Segmental Body Analyzer (Tanita, Tokyo, Japan), which employs bioelectrical impedance analysis (BIA) technology. This method uses a high-frequency electrical current and eight electrodes to measure tissue resistance, providing detailed estimates of various body composition parameters. These include body mass (kg), fat mass (FM; kg and %), fat-free mass (FFM; kg), visceral fat level (VF), percentage of muscle mass (PMM; %), phase angle (PhA; °), total body water (TBW; kg), extracellular water (ECW; kg), and intracellular water (ICW; kg). To ensure accuracy, the participants were instructed to avoid eating or drinking for at least three hours before the measurement, urinate just before the test, and refrain from alcohol consumption, excessive food or fluid intake, and intense exercise for at least 24 h prior. Individuals with implanted pacemakers or cardioverter defibrillators, as well as those with limb amputations, were excluded from this assessment due to potential measurement interference. Height was measured using a stadiometer, while waist circumference (WC), hip circumference (HC), and mid-upper arm circumference (MUAC) were recorded using a flexible, non-stretchable measuring tape. Body mass index (BMI) was calculated for all participants, along with the waist-to-hip ratio (WHR) and waist-to-height ratio (WHtR).

Peripheral BP was measured using an Omron M6 Comfort HEM-7360-E digital sphygmomanometer (Omron, Kyoto, Japan). Three readings were taken at one-minute intervals, and the average of the last two measurements was used to determine peripheral systolic and diastolic BP (pSBP and pDBP).

### 2.5. Advanced Glycation End-Product Measurement

To measure AGE in the skin, SAF, a noninvasive method with the AGE Reader mu (Diagnostic’s Technologies BV, Groningen, The Netherlands), was used. This device emits UV-A light and uses a built-in spectrometer to calculate SAF by comparing the excitation and emitted light. The results are expressed in arbitrary units (AU).

Before each measurement, we cleaned the participant’s dominant forearm with alcohol and placed it on the device, ensuring the skin area had no visible abnormalities. Each participant underwent three consecutive measurements, and we calculated the mean SAF value for accuracy. To assess CV risk, we considered SAF levels alongside the participant’s age. Using an application provided by the manufacturer, participants were classified into two CV risk categories: none and increased.

### 2.6. Statistical Analysis

Categorical data are presented with absolute and relative frequencies. Differences in categorical variables were assessed through the chi-square test. The normality of numerical variable distributions was evaluated using the Shapiro–Wilk test, and since the distribution deviates from normality, the data are presented with the median and interquartile range boundaries. To compare continuous variables between two independent groups, the Mann–Whitney U test was applied, while for three or more groups, the Kruskal–Wallis test was used, followed by the Conover post hoc test when necessary. The strength of associations is expressed using Spearman’s correlation coefficient (Rho). To determine which predictors significantly influence the outcomes, regression analysis (with adjustments) was conducted. All *p*-values are two-sided, with the significance level set at α (alpha) = 0.05. Data analysis was carried out using MedCalc^®^ Statistical Software version 23.1.7 (MedCalc Software Ltd., Ostend, Belgium; https://www.medcalc.org; 2025) [42] and SPSS version 23 [43].

## 3. Results

**Figure 2:**
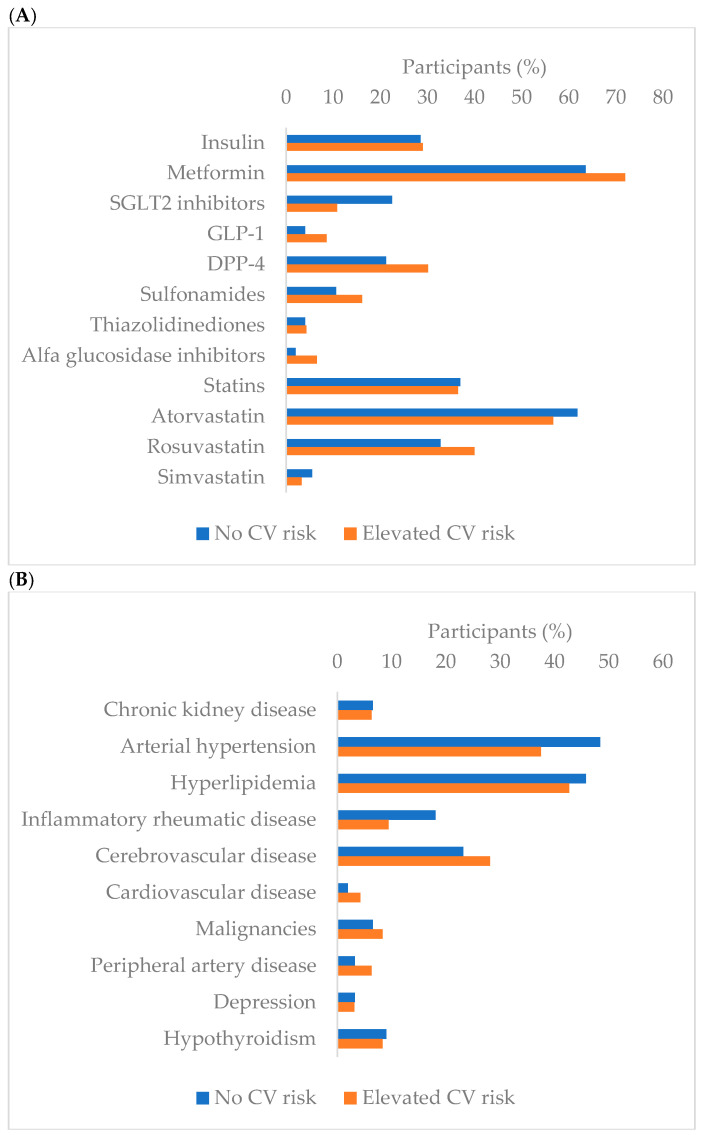
(**A**) Medication and (**B**) comorbidity stratification according to AGE-stratified CV risk.

In this study, 251 DMT2 participants were included, 130 (51.8%) women and 121 men (48.2%), with a median age of 67 years (60–73). This analysis is a sub-analysis of a previous study [40,41] where the differences in all measured parameters are explored regarding the level of CV risk according to AGE level evaluation. Participants were divided into two groups, 155 participants (61.8%) with no CV risk, and 96 participants (38.2%) with elevated CV risk according to AGE level evaluation. When observing the general characteristics (age, sex, pSBP and pDBP, smoking, duration of smoking, visits to endocrinologists and nephrologists, pharmacotherapy, and comorbidities) of these two groups, there were significantly more men in the elevated CV risk group. Therefore, pDBP was significantly higher in the elevated CV risk group, while those with no CV risk had significantly higher sodium-glucose cotransporter 2 (SGLT2) inhibitor prescriptions. Across all other parameters, the two groups were well-matched. Detailed demographic and clinical data are presented in Table 1, while medication use and comorbidities are depicted in Figure 2A,B.

### 3.1. Laboratory Parameters Regarding Cardiovascular Risk Groups

**Figure 3:**
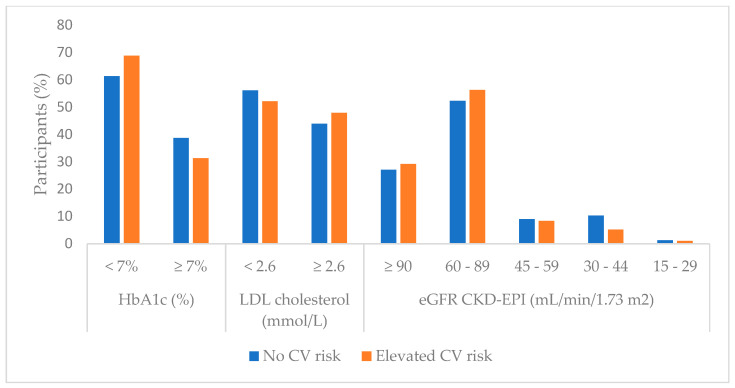
LDL cholesterol, HbA1c, and eGFR stratification.

No statistically significant differences were observed in the laboratory parameters between these two groups, except in serum creatinine values, where higher values were present in the no CV risk group (*p* = 0.04). Statistically significant laboratory findings as well as total values and differences in LDL-C role and HbA1c achieved targets and eGFR level differentiation are presented graphically in Figure 3. Complete laboratory comparisons are presented in Supplementary Table S2.

### 3.2. Anthropometric and Body Composition Measurements Regarding Cardiovascular Risk Groups

In analyzing anthropometric and body composition measurements, statistically significant differences between groups were identified only in terms of BMI and HC. The participants in the elevated CV risk group according to AGE value had a significantly higher BMI compared to the no CV risk group (27.4 vs. 27.8 kg/m^2^, *p* = 0.007). Additionally, HC was also significantly higher in the elevated CV risk group (104.5 vs. 109 cm, *p* = 0.04). All statistically significant data with BMI differentiation is shown in Table 2, while complete analysis is shown in Supplementary Table S3.

### 3.3. ABC Group Analysis

**Figure 4:**
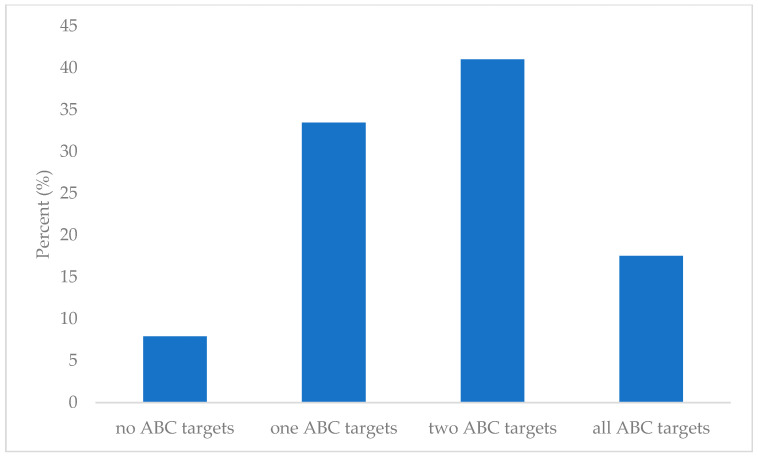
ABC stratification.

To further comprehend the CV risk in this population, the participants were divided according to achieved glycemia (A), arterial blood pressure (B), and LDL-C (C) goals. If HbA1c was lower than 7%, the “A” goal was considered achieved. If systolic blood pressure was lower than 140 mmHg, and diastolic blood pressure was lower than 90 mmHg, the “B” goal was considered achieved. If LDL-C levels were lower than 2.6 mmol/L, the “C” goal was considered achieved. A total of 20 (8%) participants had not achieved any set goals, 84 (33.5%) participants achieved only one goal, 103 (41%) participants achieved two set goals, and 44 (17.5%) participants achieved all goals. The ABC stratification of the participants is depicted in Figure 4.

**Figure 5:**
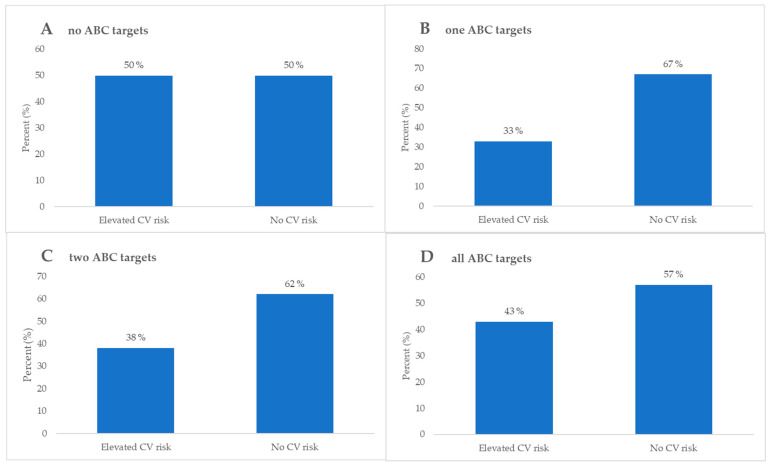
Stratification of elevated and no CV risk and comparative analysis with all participants, categorized as follows: (**A**) those who have not achieved any of the ABC targets, (**B**) those who have achieved one ABC target, (**C**) those who have achieved two ABC targets, and (**D**) those who have achieved all ABC targets.

No statistically significant differences were identified except HbA1c, blood pressure, and both LDL-C and total cholesterol values, which were all lower as more goals were achieved. Detailed analysis and differences between groups is presented in Table 3, while ABC target achievement stratified by elevated or no CV risk is depicted in Figure 5.

To further evaluate the achievement of ABC targets, participants that had prior CV or CBV incidents were analyzed separately. For these participants, “C” goals were considered achieved if LDL-C levels were lower than 1.8 mmol/L, while all other criteria remained the same. Within this group, there were 20 (30%) participants that achieved one goal, 26 (38%) participants that achieved two goals, 15 (22%) participants that achieved all goals, and 7 (10%) participants that have not achieved any goals.

**Figure 6:**
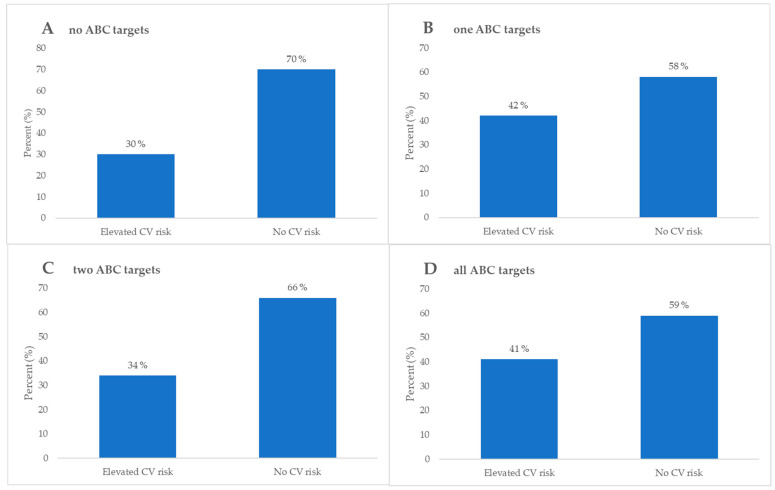
Stratification of elevated and no CV risk and comparative analysis with participants with prior CV or CBV incident categorized as follows: (**A**) those who had not achieved any of the ABC targets, (**B**) those who had achieved one ABC target, (**C**) those who had achieved two ABC targets, and (**D**) those who had achieved all ABC targets.

When observing the values of HbA1c, those who had not achieved any set goals had statistically significant higher values of HbA1c compared to those who had achieved at least one goal, while those who had achieved all goals had statistically significant lower values of HbA1c compared to those who had achieved two goals. Concurrently, total and LDL-C levels were significantly higher in those who had achieved one goal or had not achieved any goals compared to those who had achieved at least two goals. An evaluation and comparison of all parameters is presented in Table 4, while stratification according to CV risk is depicted in Figure 6.

### 3.4. Predictor Analysis

To further identify independent predictors of AGE value and number of ABC targets achieved across all participants and by participants with prior CV or CBV incidents according to medical history, a multivariance linear regression was performed.

When analyzing AGE predictors, in the multivariate model, the use of GLP1 receptor agonists was significantly associated with higher AGE values. However, the explained variance was very low (R^2^ = 0.018; adjusted R^2^ = 0.013), and the effect size was small (Cohen’s f^2^ = 0.01), indicating limited clinical relevance despite statistical significance.

When observing the number of ABC targets in all participants, although a statistically significant association was observed between sulfonamide use and the number of ABC targets achieved, the effect size was small (Cohen’s f^2^ = 0.02), and the model explained only a small proportion of outcome variance (R^2^ = 0.023; adjusted R^2^ = 0.018). These findings suggest limited clinical significance despite the statistical relevance of the association.

In the subgroup of participants with a history of CV or cerebrovascular events, the use of SGLT2 inhibitors was positively associated with the number of ABC targets achieved, while BMI was negatively associated with the outcome. The model explained 7.3% of the variance (R^2^ = 0.073; adjusted R^2^ = 0.057), with a Cohen’s f^2^ = 0.08, indicating an effect size approaching moderate.

All data and analyses are presented in Table 5.

## 4. Discussion

This single-center experience, according to demographics and clinical features, is highly consistent with findings of global diabetes research [4]. In accordance with the IDF’s latest report on the DMT2 trends, we also show a median age of mid to late 60s, nearly equal gender distribution, and a high burden of CVD [4,44]. Elevated BMI and HC are identified as the major modifiable risk factors for developing DMT2 and manifested diabetes DALYs on a worldwide scale [8,45].

In the differences between CV risk based on AGE measured by SAF, there were more men in the group of participants with higher CV risk. Interestingly, our results showed no age difference between groups by AGE-based CV risk, although AGE levels are known to naturally increase with age [46]. Nevertheless, the study by Wang et al. on 1240 subjects divided into age groups emphasizes the importance of AGE levels measured in the skin, especially in middle-aged and elderly people [47].

In terms of clinical parameters, DBP was higher in the high CV risk group based on AGE level, while SBP did not differ between groups. However, SBP is traditionally considered as important or more important than DBP for CV risk in the diabetic population [48,49]. In this context, it is important to note that only attended office BP measurement was performed, which may be influenced by various factors. In terms of laboratory values, only differences in serum creatinine levels were found, including higher values in the group without CV risk, but no significance in eGFR was found using CKD-EPI as a standardized method to estimate renal function. AGEs can trigger responses that lead to progression of KD and KD-related diseases, and they can serve as circulating biomarkers for risk stratification of KD, but this was not the case for our study sample [50].

Furthermore, no differences in LDL-C or parameters were found in relation to CV risk in our study. Although LDL-C is a known predictor of CV risk, it might not be the case when CV risk is assessed by SAF AGE levels. Nonetheless, studies with a prospective design and bigger sample size are needed [51,52]. Another interesting finding of our study is that inhibitors were prescribed almost twice as often to participants without CV risk than to participants with high CV risk, although there were no differences between the two groups in terms of HbA1c levels.

The underuse of SGLT2 inhibitors in higher-risk groups observed in our study raises clinically relevant questions about prescribing practices and clinical inertia that merit further investigation.

There are limited data suggesting the direction of the effects of SGLT-2 inhibitors on blood AGE levels [53], and more evidence is needed to understand this issue better, but this could be another pleiotropic effect of SGLT2 inhibitors.

Regarding anthropometric measurements, participants with higher CV risk based on AGE level had higher BMI and HC, both known predictive markers of increased CV risk [54,55,56]. Surprisingly, even though visceral fat tissue and fat mass in general are known contributors to increasing AGE and subsequently CV risk [57,58], body composition parameters showed no statistically significant difference between groups.

This study also reflects real-world challenges faced in the modern world of DMT2 according to the comorbidity profile depicted in both the ABC goal attainment and CV risk stratification [59]. According to the ABC target stratification in Figure 2, our cohort was suboptimally regulated since as few as 17.5% met all three ABC target values.

Despite the implementation of standardized DM care, suboptimal ABC goal attainment in our cohort likely reflects a combination of limitations in access to health care, difficulties in medication adherence, and socioeconomic factors that are particularly pronounced in the context of public health care but were not considered in this research.

The level of ABC targets met in our study is mirrored in the global rates, reported to be 10–22% achievement of optimal ABC target control in modern healthcare systems [59]. A persistent clinical challenge with the ABC protocol is compliance, possibly due to the dependence on simultaneous monitoring and control of multiple parameters [11,59]. Most of our participants met one or two targets, which is similar to the distribution pattern of ABC goal achievement in the paper by Rezaei et al. [37]. However, the extremities of ABC goal achievement are opposite for our population in comparison to theirs, indicating a selected study population that is better regulated [37]. Similarly to Rezaei et al., we can document a statistically significant correlation with increased HbA1c, total, and LDL-C in patients not achieving any ABC goals [37].

Previously, Rezaei et al. found a correlation with poorly regulated DMT2 according to the ABC targets and elevated serum AGE levels [37]. This study, focusing on skin AGE, has shown promise as a risk stratification tool in a variety of large cross-sectional studies and prospective analyses linked to metabolic syndrome, vascular disease progression, and CVD complications [60,61]. This correlation might explain the findings depicted in Figure 3, where the group that did not achieve any ABC targets had a higher likelihood of developing CV events in comparison to the group of patients reaching all the ABC targets.

To further elucidate the CV risk according to skin AF of AGE, an interesting inverse correlation of skin AGE related CV risk in patients with prior CVD is demonstrated in Figure 5. The inverse correlation was between no ABC goals achieved and an elevated risk for CV events. The literature suggests a generalizing issue with the current ABC protocol, where too tight monitoring potentially increases the risk for CVD more than the natural progression of the disease alone in the subgroup of DMT2 patients with previous CVD [62]. When comparing the findings in Figure 3 and Figure 5, there is a high risk for CV events in DMT2 patients with prior CVD who are well regulated according to the ABC protocol. The cumulative direct and indirect cardioprotective benefits of the multiple pharmacological compounds that this subgroup of patients is using are a potential explanation [63,64,65]. Furthermore, lipid-lowering and antihyperglycemic drugs have been reported in the literature to reduce skin AGE production indirectly in the early stages, since AGEs are produced by non-enzymatic glycation of sugars and lipids [66]. This highlights an inevitably evident complex interplay between DMT2, CV risk, and prior CVD that needs further attention and systematization into subgroups. Individually tailored treatment approaches might be the potential solution to this current ABC treatment protocol regimen problem.

The association between sulfonamide intake and the number of ABC targets achieved also showed very limited explanatory power and a trivial effect size. While sulfonamides may promote glycemic control by increasing insulin secretion, their overall effect on cardiometabolic management appears to be minimal. These results illustrate the difference between statistical significance and clinical relevance and should not be included in decision making in daily clinical practice, especially in the context of complex outcomes such as the achievement of ABC targets.

In contrast, SGLT2 inhibitor use was positively associated with achieving ABC goals in participants with previous CV or CBV events, while higher BMI showed a negative association. The stronger effect observed in this subgroup suggests that SGLT2 inhibitors may play a more important role in improving the cardiometabolic risk profile of high-risk individuals. This finding is consistent with existing evidence for the pleiotropic benefits of SGLT2 inhibitors, including their favorable effects on weight, BP, and CV outcomes [67,68,69].

Regression analyses revealed statistically significant associations between antidiabetic therapies and major outcomes. However, due to the limited explained variance and small effect sizes, these results should be interpreted with caution. The observed association between GLP-1 receptor agonist use and higher skin AGE levels, while statistically significant, likely reflects a marginal and clinically insignificant contribution of this therapy to tissue glycation, which should not be taken into consideration in clinical practice. This association may be influenced by residual confounding, reverse causality, or GLP-1 receptor agonist selection in patients with advanced metabolic disorders rather than a direct effect on AGE accumulation related to low prescribing volume.

Although certain therapeutic associations were statistically significant, their limited contribution to the variance in skin autofluorescence AGE and target ABC values underscores the multifactorial nature of these findings. These results underscore the importance of comprehensive, individualized treatment strategies that go beyond glucose-lowering therapies.

Since our study population was small and documented from a single center experience, our findings have reduced generalizability on a worldwide scale, even though our demographic patterns are in accordance with global reports [4]. For future directions, we encourage more studies on the same topic across ethnic groups and continents through multicentric study designs to increase the study population and increase generalizability. Furthermore, we only performed in-office BP measurements, which may be influenced by various factors, while, ideally, 24 h ambulatory BP measurement evaluation would be more suitable. In addition, a cross-sectional study design has its own natural limitations through its vast number of biases [70].

In addition, uniform HbA1c and LDL targets were used, although these are often individualized in the clinical setting based on age, comorbidities, and severity of DM complications; this may have introduced some bias. In addition, data on medical history, comorbidities, and complications were based on self-reporting by participants during screening without verification against full medical documentation, which may limit accuracy. In addition, the classification of cardiovascular risk based on AGE levels remains controversial, as it is not yet clear whether such stratification corresponds to a medium or high-risk category, which could explain the lack of differences in some variables.

Uniform ABC thresholds were deliberately applied to allow for direct comparison with previous large epidemiological studies, while acknowledging that current ADA and ESC guidelines recommend individualized targets based on patient characteristics and comorbidities, which should be considered when making decisions in clinical settings.

A suggestion for the future is to create double-blinded prospective studies on lipid-lowering and novel antihyperglycemic drugs and their effect on serum versus skin AGE CV risk reduction in patients with and without prior CVD.

## 5. Conclusions

This single-center study highlights the ongoing challenge of achieving optimal cardiometabolic control in DMT2, with fewer than one in five patients achieving all ABC targets. Our findings are consistent with global trends and emphasize the intricate interactions between glycemic management, CV risk, and prior CVD, as well as the importance of increased BMI and WC as modifiable risk factors. In patients with established CVD, the inverse association between ABC goal attainment and CV events underscores the need for more individualized interventions. Additionally, our results suggest that AGE measurement could be integrated into existing public health frameworks as a complementary screening approach, although clinical implementation requires more strong evidence from RCTs. Future prospective, multicenter studies are critical to clarify the role of innovative therapeutics in reducing AGE-related CV problems and to develop individualized treatments that go beyond glucose-centric paradigms given the multidimensional nature of cardiometabolic risk.

## Figures and Tables

**Table 1:** General characteristics of the studied population and differences between the two groups according to CV risk stratification.

General Data		No CV Risk (*n* = 155)	Elevated CV Risk (*n* = 96)	Total (*n* = 251)	*p*
Age (years), median (IQR)		68 (61–74)	67 (60–73)	67 (60–73)	0.34 ^†^
Disease duration of DMT2 ^1^ (years), median (IQR)		11 (6–20)	10 (6–20)	10 (6–20)	0.69 ^†^
Sex, *n* (%)					
Men		60 (39)	61 (64)	121 (48)	<0.001 *
Women		95 (61)	35 (36)	130 (52)	
pSBP ^1^ (mmHg), median (IQR)		138 (125–152)	140 (127.3–152)	138 (125–152)	0.34 ^†^
pDBP ^1^ (mmHg), median (IQR)		81 (75–87)	85 (76.3–91)	81 (75–88)	0.02 ^†^
Arterial BP, *n* (%)					
	<140/90 mmHg	77 (49.7)	47 (49)	124 (49.4)	0.91 *
	≥140/90 mmHg	78 (50.3)	49 (51)	127 (50.6)	
Endocrinology examination, *n* (%)		106 (68.4)	71 (74)	177 (70.5)	0.35 *
Nephrology examination, *n* (%)		54 (34.8)	26 (27.1)	80 (31.9)	0.20 *
Regular nephrology examination, *n* (%)		19 (12.3)	12 (12.5)	31 (12.4)	0.96 *
Smoking, *n* (%)		28 (18.1)	16 (16.7)	44 (17.5)	0.78 *
Duration of smoking, median (IQR)		30 (25–40)	38 (30–47)	30 (25–44)	0.77 ^†^
Former smokers, *n* (%)		54 (34.8)	32 (33.3)	86 (34.3)	0.81 *
Time without smoking (years), median (IQR)		15 (6–23)	15 (11–29)	15 (8–25)	0.69 ^†^

* χ^2^ test; ^†^ Mann–Whitney U test; ^1^ Abbreviations: DMT2—diabetes mellitus type 2, pSBP—peripheral systolic blood pressure, pDBP—peripheral diastolic blood pressure, BP—blood pressure, SGLT2—sodium/glucose cotransporter 2, GLP-1—glucagon-like peptide-1, DPP-4—dipeptidyl peptidase-4, CKD—chronic kidney disease, AH—arterial hypertension, CBD—cerebrovascular disease, CVD—cardiovascular disease, PAD—peripheral artery disease.

**Table 2:** Statistically significant differences in anthropometric measurements (with BMI differentiation) between the two groups according to CV risk stratification.

		No CV Risk (*n* = 155)	Elevated CV Risk (*n* = 96)	Total (*n* = 251)	*p* *
BMI ^1^ (kg/m^2^), median (IQR)		27.4 (23.8–30.6)	27.8 (24.4–32.9)	27.3 (22.2–31.6)	0.007
	<25 kg/m^2^, *n* (%)	48 (31.6)	26 (27.7)	74 (30.1)	0.67 ^†^
	≥25 kg/m^2^ and <30 kg/m^2^, *n* (%)	60 (39.5)	36 (38.3)	96 (39)	
	≥30 kg/m^2^, *n* (%)	44 (28.9)	32 (34)	76 (30.9)	
HC ^1^ (cm), median (IQR)		104.5 (99–113)	109 (102.5–115)	108 (99–113)	0.04

* Mann–Whitney U test; ^†^ χ^2^ test; ^1^ Abbreviations: BMI—body mass index, HC—hip circumference.

**Table 3:** Achieved ABC goal stratification and comparison of main parameters.

		No Goals Achieved (*n* = 20)	One Goal Achieved (*n* = 84)	Two Goals Achieved (*n* = 103)	All Goals Achieved (*n* = 44)	*p*
Age (years), median (IQR)		64 (59–68)	68 (61–74)	68 (59–74)	67 (63–71)	0.34 ^†^
Sex, *n* (%)						
	Men	11 (55)	50 (48.5)	42 (50)	18 (40.9)	0.70
	Women	9 (45)	53 (51.5)	42 (50)	26 (59.1)	
Disease duration of DMT2 (years), median (IQR)		18 (11–23)	10 (4–20)	12 (6–20)	10 (7–20)	0.07 ^†^
pSBP ^1^ (mmHg), median (IQR)		149 (144–164)	150 (137–160)	135 (125–145)	122 (114–133)	<0.001 ^†‡^
pDBP ^1^ (mmHg), median (IQR)		86 (93–90)	87 (81–94)	80 (74–86)	76 (72–84)	<0.001 ^†‡^
BMI ^1^ (kg/m^2^), median (IQR)		27.5 (23.7–31.3)	27.4 (23.9–31.8)	27.9 (24.7–30.8)	27.0 (23.0–30.5)	0.85 ^†^
WHR ^1^, median (IQR)		0.96 (0.90–0.99)	0.94 (0.87–0.98)	0.93 (0.87–0.99)	0.92 (0.88–0.98)	0.72 ^†^
eGFR CKD-EPI ^1^ (mL/min/1.73 m^2^), median (IQR)		74.1 (54.4–90.2)	76.3 (65.7–93.9)	78 (65.7–90.9)	75.9 (60.7–88.2)	0.48 ^†^
HbA1c ^1^ (%), median (IQR)		7.5 (7.3–8.0)	8 (6.4–8.1)	6.5 (6.0–7.0)	6.2 (5.8–6.7)	<0.001 ^†‡^
AGE ^1^, median (IQR)		2.4 (1.9–2.9)	2.2 (1.9–2.7)	2.2 (2.0–2.6)	2.3 (1.9–2.9)	0.71 ^†^
	Elevated CV ^1^ risk, *n* (%)	10 (50)	56 (67)	64 (62)	25 (57)	0.48 *
	No CV ^1^ risk, *n* (%)	10 (50)	28 (33)	39 (38)	19 (43)	
Total cholesterol (mmol/L), median (IQR)		5.7 (5.2–6.3)	5.2 (4.5–5.9)	4.4 (3.7–5.1)	3.75 (3.35–4.35)	<0.001 ^†‡^
HDL ^1^ cholesterol (mmol/L), median (IQR)		1.35 (1.2–1.5)	1.5 (1.3–1.7)	1.5 (1.2–1.7)	1.4 (1.1–1.6)	0.69 ^†^
LDL ^1^ cholesterol (mmol/L), median (IQR)		3.3 (2.9–3.7)	2.9 (2.4–3.5)	2.1 (1.7–2.9)	1.8 (1.4–2.0)	<0.001 ^†‡^
Triglycerides (mmol/L), median (IQR)		1.9 (1.35–2.45)	1.4 (1.05–2.1)	1.3 (0.9–2.0)	1.3 (0.85–1.75)	0.05 ^†^
Albuminuria (mg/L), median (IQR)		5.5 (4–12)	5 (2–17)	5.0 (2.0–13)	5.5 (52–9.5)	0.86 ^†^

* χ^2^ test; ^†^ Kruskal–Wallis test (post hoc test: Conover); ^‡^ all groups are statistically significantly different. ^1^ Abbreviations: DMT2—diabetes mellitus type 2, pSBP—peripheral systolic blood pressure, pDBP—peripheral diastolic blood pressure, BMI—body mass index, WHR—waist-to-hip ratio, eGFR CKD-EPI—estimated glomerular filtration ratio using Chronic Kidney Disease Epidemiology Collaboration, AGE—advanced glycation end-products, HbA1c—hemoglobin A1c, HDL—high-density lipoprotein, LDL—low-density lipoprotein.

**Table 4:** Achieved ABC goal stratification and comparison of main parameters in participants with prior CV or CBV incidents.

		No Goals Achieved (*n* = 7)	One Goal Achieved (*n* = 20)	Two Goals Achieved (*n* = 26)	All Goals Achieved (*n* = 15)	*p* ^†^
Age (years), median (IQR)		72 (63–77)	71 (62.8–76)	66 (57.8–71.3)	70 (62–73)	0.28
Sex, *n* (%)						
	Men	5 (71.4)	9 (45)	17 (65.4)	9 (60)	0.51 *
	Women	2 (28.6)	11 (55)	9 (34.6)	6 (40)	
Disease duration of DMT2 ^1^ (years), median (IQR)		20 (10–26)	10.5 (10–26.5)	10 (3.8–19.3)	14 (7.5–30)	0.28
pSBP ^1^ (mmHg), median (IQR)		143 (131–146)	140.5 (127.3–155.8)	138 (130.5–149.3)	129 (121–145)	0.31
pDBP ^1^ (mmHg), median (IQR)		85 (78–88)	78 (74.3–84.3)	79 (73.8–88)	80 (74–84)	0.42
BMI ^1^ (kg/m^2^), median (IQR)		31.5 (26.7–34.9)	28.4 (24.2–30)	27.9 (23.7–31.5)	25.7 (22.7–28.1)	0.15
WHR ^1^, median (IQR)		1 (1–1.1)	0.9 (0.9–1)	0.9 (0.9–1)	1 (0.9–1)	0.14
eGFR CKD-EPI ^1^ (mL/min/1.73 m^2^), median (IQR)		72.5 (54.1–75.3)	69.3 (60.5–82.5)	65 (57.1–82.9)	75.4 (62.8–84.3)	0.87
HbA1c ^1^ (%), median (IQR)		7.8 (7.3–9.4)	6.6 (5.8–7.9)	6.9 (6.2–7.4)	6.4 (5.8–6.7)	0.003 ^‡^
AGE ^1^, median (IQR)		2.8 (2.7–3.1)	2.3 (2–2.6)	2.2 (1.9–2.6)	2.2 (2–3.1)	0.17
	Elevated CV ^1^ risk, *n* (%)	7 (100)	13 (65)	21 (81)	12 (80)	0.29 *
	No CV ^1^ risk, *n* (%)	0 (0)	7 (35)	5 (19)	3 (20)	
Total cholesterol (mmol/L), median (IQR)		5.2 (4.9–6.1)	5.0 (4.2–5.9)	3.9 (3.5–4.3)	3.4 (3–3.6)	<0.001 ^║^
HDL ^1^ cholesterol (mmol/L), median (IQR)		1.4 (1.3–1.8)	1.5 (1.1–1.6)	1.4 (1.1–1.6)	1.4 (1.3–1.6)	0.97
LDL ^1^ cholesterol (mmol/L), median (IQR)		3.0 (2.9–3.5)	2.8 (2.2–3.3)	1.8 (1.4–2.1)	1.4 (1–1.5)	<0.001 ^║^
Triglycerides (mmol/L), median (IQR)		1.8 (0.9–2.1)	1.6 (1.1–2.2)	1.3 (0.9–1.7)	1.2 (0.9–1.6)	0.18
Albuminuria (mg/L), median (IQR)		8 (5–17)	10.5 (2–44.5)	5.5 (2–13.5)	5 (2–6)	0.14

* Fisher exact test; ^†^ Kruskal–Wallis test (post hoc test: Conover); ^‡^ *p* < 0.05 significance is found between none goals group and all other groups, and between two goals and all achieved goals group ^║^ *p* < 0.05 significance is found between none/one goals achieved and two/all goals achieved groups; ^1^ Abbreviations: DMT2—diabetes mellitus type 2, pSBP—peripheral systolic blood pressure, pDBP—peripheral diastolic blood pressure, BMI—body mass index, WHR—waist-to-hip ratio, eGFR CKD-EPI—estimated glomerular filtration ratio using Chronic Kidney Disease Epidemiology Collaboration, AGE—advanced glycation end-products, CV—cardiovascular, HbA1c—hemoglobin A1c, HDL—high-density lipoprotein, LDL—low-density lipoprotein.

**Table 5:** The influence of independent predictors on AGE value and number of ABC targets achieved (all participants and only high-risk participants, multivariate linear regression—Stepwise method).

	ß	*p* Value	95% CI	Regression Module
AGE *				
GLP1 *	0.31	0.04	0.006–0.619	R^2^ = 0.018; R^2^_adj_ = 0.013 F_(1, 222)_ = 4.03; *p* = 0.04 Cohen’s f^2^ = 0.01
Constant	2.31	<0.001	2.24–2.38	
**Number of ABC targets in all participants**				
Sulfonamides	−0.39	0.02	−0.72–−0.05	R^2^ = 0.023; R^2^_adj_ = 0.018 F_(1, 222)_ = 5.15; *p* = 0.02 Cohen’s f^2^ = 0.02
Constant	1.73	<0.001	1.61–1.85	
Number of ABC targets in participants with prior CV or CBV incidents *				
SGLT2 *	0.69	0.02	0.10–1.29	R^2^ = 0.073; R^2^_adj_ = 0.057 F_(2, 57)_ = 4.71; *p* = 0.01 Cohen’s f^2^ = 0.08
BMI *	−0.05	0.03	−0.09–−0.003	
Constant	2.88	<0.001	1.65–4.09	

ß—regression coefficient; * Abbreviations: AGE—advanced glycation end-products, GLP-1—glucagon-like peptide-1, CV—cardiovascular, CBV—cerebrovascular, SGLT2—sodium/glucose cotransporter 2, BMI—body mass index.

## Data Availability

All data is available upon reasonable request to the corresponding author.

## Supplementary Materials

The following supporting information can be downloaded at: https://www.mdpi.com/article/10.3390/biomedicines13102418/s1. Table S1: Comparison of uniform ABC thresholds with current individualized guideline targets; Table S2: Differences in laboratory parameters according to cardiovascular risk; Table S3: Differences in anthropometric measurements (with BMI differentiation) between two groups according to CV risk stratification. References [71,72,73] are cited in the supplementary materials.
