# Supplementary material for: Public Health Screening for Cardiometabolic Risk: Lessons from Advanced Glycation End-Products and ABC Target Achievement in Dalmatian Adults with Type 2 Diabetes
Source: Biomedicines. 2025 Oct 2;13(10):2418. doi: 10.3390/biomedicines13102418 (PMC12561152; doi:10.3390/biomedicines13102418)
Supplement: Supplementary file 1 [file biomedicines-13-02418-s001.zip › biomedicines-3850931-supplementary.pdf]

Supplementary Table S1. Comparison of Uniform ABC Thresholds with Current Individualized Guideline Targets

**Parameter**	[1] **ABC Threshold Used in Study**	**Current Individualized Guideline Targets***	**Key Notes**
[1] **HbA1c**	< 7.0 %	< **6.5 %** for healthy adults with low hypoglycemia risk; < **7.0 %** for most adults; < **7.5 %** for healthy older adults; < **8.0 %** for older adults with complex/intermediate health; No [1]HbA1C goal for very complex/poor health or limited life expectancy	Individualize based on age, comorbidities, cognitive impairment and treatment burden
**Blood Pressure**	< 140/90 mmHg	General: first objective of treatment < 140/90 mmHg and if well tolerated treat to ≤ 130/80 mmHg if tolerated; consider 120–129 mmHg systolic for further [1]CV risk reduction; in older/frail patients target 130–139 mmHg systolic and < 80 mmHg diastolic	[1] ESC 2024: personalize for orthostatic hypotension, frailty, or limited life expectancy
[1] **LDL-C**	< 2.6 mmol/L	Low risk: < **3.0 mmol/L**; Moderate risk: < **2.6 mmol/L**; High risk: < **1.8 mmol/L**; Very high risk: < **1.4 mmol/L**; Extreme risk (selected): < **1.0 mmol/L**	Also recommend ≥50% reduction from baseline for high/very high risk

[1] Abbreviations: ABC – HbA1c, Blood Pressure, LDL-C composite target; ADA – American Diabetes Association; HbA1C – Glycated Hemoglobin; CV – Cardiovascular; ESC – European Society of Cardiology; EAS – European Atherosclerosis Society; HbA1c – Hemoglobin A1c; LDL-C – Low-Density Lipoprotein Cholesterol

*Sources: ADA Standards of Care 2025; ESC Hypertension Guidelines 2024; ESC/EAS Dyslipidemia Guidelines Update 2025 [71–73]

Supplementary Table S2. Differences in laboratory parameters according to cardiovascular risk.

	**No CV risk (n=155)**	**Elevated CV risk (n=96)**	**Total (n=251)**	***P****
WBC[1] (x10^9/L), median (IQR)	7 (5.8 – 8.4)	6.8 (5.7 – 7.9)	6.8 (5.8 – 8.1)	0.46
RBC[1], median (IQR)	5 (4.3 - 5)	4.7 (4.4 - 5)	4.7 (4.37 – 4.99)	0.93
Hb[1] (g/L), median (IQR)	141 (127 - 152)	140.5 (128 – 150.8)	141 (128 - 151)	0.77
Htc[1] (L/L), median (IQR)	414 (380 - 446)	411.5 (383.3 - 435)	413 (381.25 - 441)	0.61
MCV[1] (fL), median (IQR)	89 (86 – 91.1)	88,1 (85.6 – 90.2)	88.35 (86 - 91)	0.39
MCH[1] (pg), median (IQR)	31 (29.1 – 31.4)	30 (29 – 31.1)	30.3 (29.03 – 31.4)	0.39
MCHC[1] (g/L), median (IQR)	340 (334 - 349)	342 (336 – 347.8)	342 (335 – 348.75)	0.72
RDW[1] (%), median (IQR)	13 (12.4 – 13.6)	13 (12.5 – 13.5)	13 (12.5 – 13.5)	0.58
Platelet count (x10^9/L), median (IQR)	240 (196 - 287)	235.5 (199.5 – 269.8)	238 (198.25 – 280.5)	0.48
Neutrophiles (%), median (IQR)	60 (53.1 – 65.7)	58.5 (52.9 – 64.5)	59.25 (52.95 – 65.13)	0.46
Lymphocytes (%), median (IQR)	29 (24.3 – 34.4)	30.2 (24 – 37.5)	29.2 (24.15 – 35.3)	0.27

Monocytes (%), median (IQR)	8 (6.7 – 9.2)	7.7 (6.4 – 8.6)	7.85 (6.6 – 8.9)	0.09
Eosinophiles (%), median (IQR)	2 (1.5 – 3.4)	2.1 (1.4 – 3.7)	2.2 (1.43 – 3.4)	0.75
Basophiles (%), median (IQR)	1 (0.5 – 0.8)	0.7 (0.5 – 0.8)	0.6 (0.5 – 0.8)	0.37
Glucose (mmol/L), median (IQR)	7 (5.8 – 9.3)	6.8 (5.6 - 9)	6.9 (5.7 – 9.08)	0.69
Creatinine (mmol/L), median (IQR)	83 (71 - 100)	78 (65.3 - 91)	81 (69 - 96)	**0.04**
HbA1c[1] (%), median (IQR)	7 (6.2 – 7.3)	6.7 (6.1 – 7.1)	6.7 (6.1 – 7.3)	0.46
< 7%, *n* (%)	95 (61.3)	66 (68.8)	161 (64.1)	0.23†
≥ 7%, *n* (%)	60 (38.7)	30 (31.3)	90 (35.9)	
Creatinine (mg/dU), median (IQR)	7 (4.5 – 9.6)	6.7 (4 – 9.8)	6.78 (4.44 – 9.61)	0.51
Albuminuria (mg/dU), median (IQR)	5 (2 - 14)	5 (2 – 12.5)	5 (2 - 13)	0.32
ACR[1] (mg/mmol), median (IQR)	1 (0.5 – 1.7)	0.6 (0.4 – 1.8)	0.75 (0.46 – 1.73)	0.39
Tg[1] (mmol/L), median (IQR)	1 (1 – 2.1)	1.3 (0.9 - 2)	1.4 (0.9 – 2)	0.40
HDL[1] cholesterol (mmol/L), median (IQR)	1 (1.2 – 1.6)	1.5 (1.2 – 1.8)	1.4 (1.2 – 1.7)	0.36
Total cholesterol (mmol/L), median (IQR)	5 (3.7 – 5.4)	4.7 (4 – 5.6)	4.6 (3.8 – 5.5)	0.34
LDL[1] cholesterol (mmol/L), median (IQR)	2 (1.7 – 3.1)	2.5 (1.9 – 3.1)	2.4 (1.8 – 3.1)	0.30
< 2.6, *n* (%)	87 (56.1)	50 (52.1)	137 (54.6)	0.53†
≥ 2.6, *n* (%)	68 (43.9)	46 (47.9)	114 (45.4)	
eGFR CKD-EPI[1] (mL/min/1.73 m²), median (IQR)	75 (61.2 – 91.2)	78.4 (66.9 – 92.2)	77 (62.7 – 91.2)	
≥ 90	42 (27.1)	28 (29.2)	70 (27.9)	
60 – 89	81 (52.3)	54 (56.3)	135 (53.8)	
45 – 59	14 (9)	8 (8.3)	22 (8.8)	0.70†
30 – 44	16 (10.3)	5 (5.2)	21 (8.4)	
15 – 29	2 (1.3)	1 (1)	3 (1.2)	

*Mann-Whitney U test; †χ^2 test

[1] Abbreviations: WBC - leukocytes, RBC - red blood cell count, MCV - mean corpuscular volume, Hb - hemoglobin, MCH - mean cellular hemoglobin, MCHC - RDW - red cell distribution width, MPV - mean platelet volume, eGFR CKD-EPI - estimated glomerular filtration ratio using Chronic Kidney Disease Epidemiology Collaboration, Tg - triglycerides, HDL - high density lipoprotein, LDL - low density lipoprotein, HbA1c - hemoglobin A1c, ACR - albumin-to-creatinine ratio.

Supplementary Table S3. Differences in anthropometric measurements (with BMI differentiation) between two groups according to CV risk stratification.

	**No CV risk (n=155)**	**Elevated CV risk (n=96)**	**Total (n=251)**	***P****
BMI[1] (kg/m²), median (IQR)	27.4 (23.8 – 30.6)	27.8 (24.4 – 32.9)	27.3 (22.2 – 31.6)	**0.007**
< 25 kg/m², *n* (%)	48 (31.6)	26 (27.7)	74 (30.1)	
≥ 25 kg/m² and < 30 kg/m², *n* (%)	60 (39.5)	36 (38.3)	96 (39)	0.67†
≥ 30 kg/m², *n* (%)	44 (28.9)	32 (34)	76 (30.9)	
Height (cm), median (IQR)	172 (164 – 179.8)	173 (165.8 - 180)	174 (165 - 179)	0.26
Weight (kg), median (IQR)	79.9 (69.4 – 94.3)	85.8 (72.1 – 95.4)	81.1 (65.9 - 94)	0.14
MUAC[1] (cm), median (IQR)	30 (27 - 32)	30 (27.8 - 32)	30 (27 - 32)	0.34
WC[1] (cm), median (IQR)	98 (88 - 110)	104 (94 - 113)	98 (90 - 110)	0.07
HC[1] (cm), median (IQR)	104.5 (99 - 113)	109 (102.5 - 115)	108 (99 - 113)	**0.04**

WHR[1], median (IQR)	0.9 (0.9 - 1)	0.9 (0.9 - 1)	0.94 (0.87 - 1)	0.67
FM[1] (%), median (IQR)	30 (22.9 – 35.6)	30.4 (23.3 – 37.9)	30.1 (23.1 – 36.5)	0.29
FM[1] (kg), median (IQR)	23 (17.8 – 30.6)	25 (17.9 - 35)	23.6 (17.9 – 31.8)	0.24
PhA[1], median (IQR)	5 (4.8 – 5.8)	5.3 (4.8 - 6)	5.4 (4.8 – 5.9)	0.86
VF[1] level, median (IQR)	11 (9 - 14)	11 (9 - 14)	11 (9 - 14)	0.93
FFM[1] (kg), median (IQR)	56 (48.6 – 66.9)	57.9 (47.9 – 69.3)	56.8 (48.4 – 68.2)	0.79
TBW[1] (kg), median (IQR)	39 (34.4 – 46.6)	40.3 (34.2 – 47.4)	39.6 (34.2 – 46.7)	0.76
ECW[1] (kg), median (IQR)	18 (15.9 – 20.3)	18.5 (15 – 20.6)	18.1 (15.8 – 20.4)	0.61
ICW[1] (kg), median (IQR)	22 (18.5 – 27.1)	21.8 (18.7 – 27.1)	21.7 (18.6 – 27.1)	0.97
PMM[1] (%), median (IQR)	54 (46.1 – 63.6)	55 (45.5 – 65.9)	53.9 (45.9 – 64.8)	0.76

*Mann-Whitney U test; †χ^2 test

[1] Abbreviations: BMI - body mass index, WHR – waist-to-height ratio, WC - waist circumference, HC - hip circumference, MUAC - mid-upper arm circumference, FM - fat mass, PhA - phase angle, VF - visceral fat, FFM – fat-free mass, TBW - total body water, ECW - extracellular water, ICW - intracelular water, PMM - percentage of muscle mass.
